# β-adrenergic receptor activation in immortalized human urothelial cells stimulates inflammatory responses by PKA-independent mechanisms

**DOI:** 10.1186/1478-811X-3-10

**Published:** 2005-08-09

**Authors:** Erin B Harmon, Jill M Porter, James E Porter

**Affiliations:** 1 Department of Pharmacology, Physiology, and Therapeutics; University of North Dakota; School of Medicine & Health Sciences; Grand Forks, ND 58202-9037, USA

## Abstract

**Background:**

Interstitial cystitis (IC) is a debilitating disease characterized by chronic inflammation of the urinary bladder, yet specific cellular mechanisms of inflammation in IC are largely unknown. Multiple lines of evidence suggest that β-adrenergic receptor (AR) signaling is increased in the inflamed urothelium, however the precise effects of these urothelial cell signals have not been studied. In order to better elucidate the AR signaling mechanisms of inflammation associated with IC, we have examined the effects of β-AR stimulation in an immortalized human urothelial cell line (UROtsa). For these studies, UROtsa cells were treated with effective concentrations of the selective β-AR agonist isoproterenol, in the absence or presence of selective inhibitors of protein kinase A (PKA). Cell lysates were analyzed by radioimmunoassay for generation of cAMP or by Western blotting for induction of protein products associated with inflammatory responses.

**Results:**

Radioligand binding demonstrated the presence of β-ARs on human urothelial UROtsa cell membranes. Stimulating UROtsa cells with isoproterenol led to concentration-dependent increases of cAMP production that could be inhibited by pretreatment with a blocking concentration of the selective β-AR antagonist propranolol. In addition, isoproterenol activation of these same cells led to significant increases in the amount of phosphorylated extracellular signal-regulated kinase (pERK), inducible nitric oxide synthase (iNOS) and the induced form of cyclooxygenase (COX-2) when compared to control. Moreover, preincubation of UROtsa cells with the selective PKA inhibitors H-89 or Rp-cAMPs did not diminish this isoproterenol mediated phosphorylation of ERK or production of iNOS and COX-2.

**Conclusion:**

Functional β-ARs expressed on human urothelial UROtsa cell membranes increase the generation of cAMP and production of protein products associated with inflammation when activated by the selective β-AR agonist isoproterenol. However, the increased production of iNOS and COX-2 by isoproterenol is not blocked when UROtsa cells are preincubated with inhibitors of PKA. Therefore, UROtsa cell β-AR activation significantly increases the amount of iNOS and COX-2 produced by a PKA-independent mechanism. Consequently, this immortalized human urothelial cell line can be useful in characterizing potential AR signaling mechanisms associated with chronic inflammatory diseases of the bladder.

## Background

Interstitial cystitis (IC) is a debilitating disease characterized by chronic pain in the urinary bladder along with increased urinary frequency and urgency. IC is a complex disease with multiple etiologies, yet inflammatory pain is a common mechanism of all IC symptoms [1]. Prostanoids, arachidonic acid metabolites of the cyclooxygenase (COX) pathway, and nitric oxide (NO), whose formation is catalyzed by nitric oxide synthase (NOS), both play major roles in regulating the inflammatory response. Increased levels of prostaglandins generated by the inducible form of cyclooxygenase (COX-2) mediate the vasodilatation and vascular permeability observed during the early events of inflammation [2]. Moreover, animal models lacking the PGE2 prostaglandin receptor demonstrate a reduced algesic response indicating the importance of prostanoids in the signaling and perception of inflammatory pain [3]. Finally, increased COX-2 expression documented for an *in vivo *model of cystitis supports the idea that increased prostaglandin signaling sensitizes bladder afferents that control micturition and pain [4].

The expression of the inducible form of NOS, iNOS, has been characterized in numerous cell types as a consequence of the inflammatory processes that follow tissue damage [5]. Large amounts of NO generated by iNOS surpass homeostatic concentrations formed by endothelial eNOS or neuronal nNOS [6]. This difference in kinetics of NO formation by iNOS leads to multiple inflammatory responses that include neutrophil activation, DNA damage, protein nitration and induction of apoptosis [5]. Furthermore, animal models deficient in iNOS establish this enzyme's importance as a pathophysiological mediator of chronic inflammatory diseases [7]. Moreover, increased levels of luminal NO, recognized as a causative agent for bladder excitability and micturition, has been documented in patients with IC, which could represent a mechanism of hyperexcitability documented for this disease [8].

Multiple lines of evidence suggest that increased signaling through the G protein-coupled β-adrenergic receptor (AR) may be linked to inflammation associated with IC. Patients with IC have been found to have increased nerve fiber innervation of the urinary bladder. Further study has shown these fibers to be solely sympathetic nerves, which would correspond to an increase in AR signaling [9]. Moreover, elevated urinary levels of norepinephrine have been found in IC patients, which is also consistent with greater AR activity in the urinary bladder [10]. Finally, genomic profiling found increased transcription of the β_2_-AR gene in a mouse bladder inflammation model [11]. Together, these observations suggest that chronic β-AR stimulation may be linked to inflammatory bladder diseases like IC. Therefore, we hypothesize that urothelial β-AR activation mediates specific inflammatory responses that can be linked to bladder hyperexcitability and pain documented in chronic inflammatory bladder diseases like IC.

In order to test this hypothesis, we have studied the effects of β-AR activation in an immortalized cell line of human urothelium, UROtsa cells [12]. UROtsa cells exhibit numerous properties of basal bladder epithelial cells, including the potential to differentiate into the stratified cell types found in the mammalian bladder lining. Our results using these UROtsa cells as an *in vitro *model of bladder urothelium, reveals a correlation between β-AR activation and the production of specific pro-inflammatory proteins via a PKA-independent mechanism.

## Results

### Identification of specific β-AR binding sites

**Figure 1:**
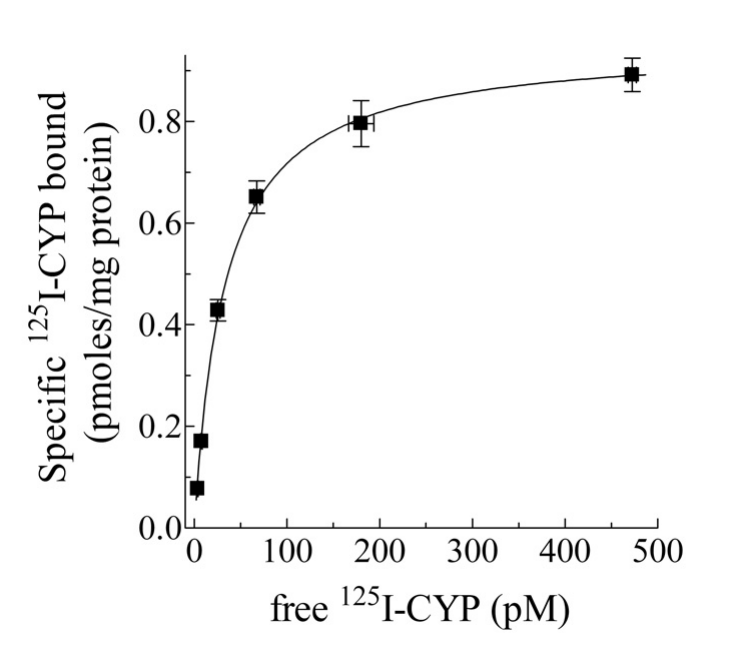
**Specific β-AR binding sites expressed on UROtsa cell membranes**. UROtsa cell membranes were incubated with increasing amounts of ^125^I-CYP in the absence and presence of 10 μM propranolol to determine total and nonspecific binding, respectively. Specific ^125^I-CYP binding was plotted versus the amount of free radioligand concentration and fitted to a rectangular hyperbola using non-linear regression analysis. From this curve fit the number of specific ^125^I-CYP binding sites was determined to be 951 ± 43 fmol/mg protein. In addition, the equilibrium dissociation constant of ^125^I-CYP for these binding sites was calculated to be 32.4 ± 1.1 pM. Values are presented as the mean ± S.E. for *n *= 3 experiments performed in duplicate.

It was important to determine if specific AR binding sites are expressed on UROtsa cell membranes in order to study specific inflammatory responses that may be linked to β-AR activation. Therefore, radioligand binding analysis was performed to characterize the level of β-AR expression on UROtsa cell membranes. Increasing amounts of the iodinated non-selective β-AR antagonist (-)3-[^125^I]iodocyanopindolol (^125^I-CYP) specifically labeled a saturable, homogeneous, high affinity binding site on these UROtsa membranes (Figure 1). The number of binding sites was determined to be 951 ± 43 fmol/mg protein (*n *= 3), and the equilibrium dissociation constant of ^125^I-CYP for these binding sites was 32.4 ± 1.1 pM (*n *= 3). This ^125^I-CYP equilibrium dissociation constant is similar to the calculated affinity value of this radiochemical when it was used by others to identify β-AR binding sites on cell membranes [13].

### Isoproterenol Induces cAMP Accumulation

**Figure 2:**
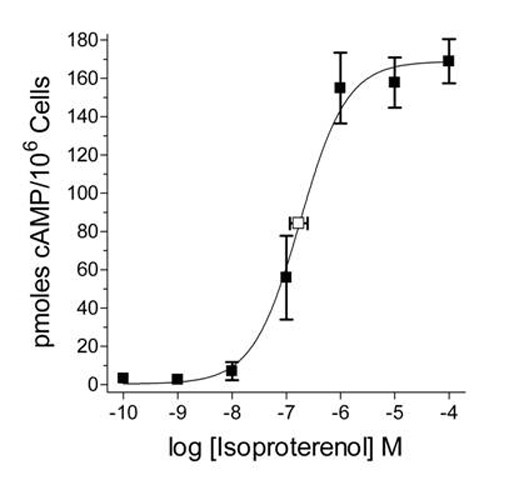
**β-AR activation stimulates cAMP production in UROtsa cells**. UROtsa cells incubated for 30 min with the selective β-AR agonist isoproterenol generated increasing amounts of cAMP in a concentration-dependent manner. From these data the concentration of isoproterenol that generated a half-maximal response (EC_50_) was calculated using non-linear regression analysis. The EC_50 _value (ϒ) of isoproterenol to increase cAMP in UROtsa cells was calculated to be 170 ± 66 nM and is presented as the mean ± S.E. for *n *= 4 experiments performed in duplicate.

β-AR activation is classically linked to adenylate cyclase activation. Therefore, to test the functionality of β-ARs expresssed on UROtsa cells, we analyzed levels of cAMP generated after incubation with a selective β-AR agonist. Isoproterenol induced a concentration-dependent increase of cAMP in UROtsa cells (Figure 2). From this data a concentration-response curve was generated and used to calculate a half-maximal isoproterenol concentration (EC_50_) for generating cAMP in these cells. The calculated EC_50 _of 170 ± 66 nM (*n *= 4) for generating cAMP in these cells is similar to results by others when isoproterenol was used to stimulate β-AR production of cAMP [13]. In addition, this UROtsa cell response was abated to basal levels by preincubation with 1 μM of the selective β-AR antagonist propranolol (data not shown). These results together demonstrate that β-ARs are expressed on UROtsa cells and can be stimulated by selective receptor agonists to generate cAMP, which is characteristically linked to this AR type.

### Selective Production of Inflammatory Mediators by β-AR Stimulation

**Figure 3:**
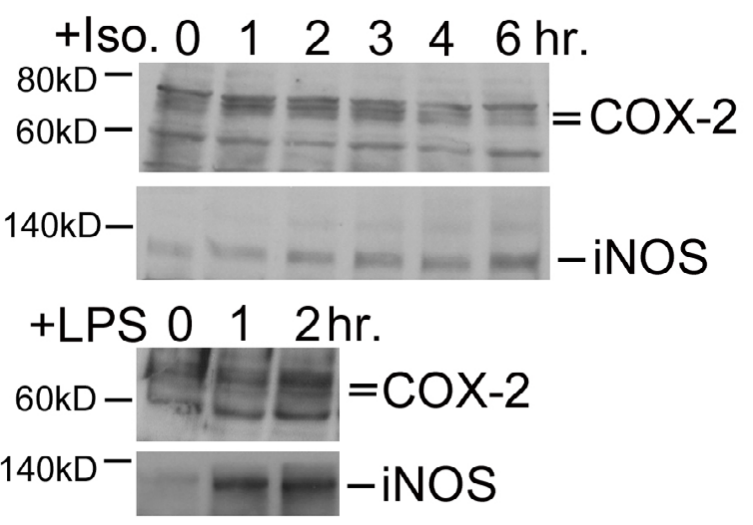
**UROtsa cell β-AR activation stimulates production of pro-inflammatory mediators**. UROtsa cells were incubated with 100 nM isoproterenol or 300 nM lipopolysaccharide (LPS) in serum-free DMEM for the indicated times. Cells were lysed in modified RIPA and total cell lysates were processed as described under "Methods" for immunoblotting with anti-COX-2 or anti-iNOS antibody to determine β-AR mediated changes in protein expression. As reported by the antibody supplier, COX-2 is identified as a doublet band running at 72–74 kD, and iNOS is detected as a band at 130 kD. Peak expression of the pro-inflammatory mediators COX-2 and iNOS was observed 2 hrs after addition of isoproterenol. Semi-quantitative analysis revealed that there was a significant 1.8 ± 0.3 and 2.1 ± 0.3 fold increase in COX-2 and iNOS expression, respectively when compared to basal. Similarly, a 2 hr incubation with LPS led to increased expression of COX-2 and iNOS when compared to basal. Values are presented as the mean ± S.E. and the autoradiographs are representative immunoblots of *n *= 3–9 independent UROtsa cell treatments.

Increased sympathetic innervation and elevated urinary catecholamine levels in IC patients implies that chronic bladder inflammation may be linked to stimulation of β-ARs. Therefore, we stimulated UROtsa cells with an effective concentration (100 nM) of isoproterenol and probed the cell lysates for mediators of inflammatory responses using Western analysis. Semi-qualitative analysis demonstrated a significant increase in protein levels for COX-2 and iNOS 2 hr after addition of isoproterenol (Figure 3). Specifically there was a 1.8 ± 0.3 and 2.1 ± 0.3 fold increase in COX-2 (*n *= 8) and iNOS (*n *= 9) expression, respectively when compared to basal. Conversely, isoproterenol treatment did not raise levels of the inflammatory cytokines IL-1β, IL-10, and IL-8 (data not shown). In separate experiments, UROtsa cells were also treated with a nonspecific agent of inflammation in order to confirm the inflammatory nature of protein expression observed after isoproterenol addition. Stimulation of UROtsa cells with 300 nM lipopolysaccharide (LPS) induced the expression of both COX-2 and iNOS (*n *= 3; Figure 3). Maximum expression of COX-2 and iNOS was observed 2 hr after LPS addition, which is similar to the time course observed using isoproterenol. These results demonstrate a selective production of inflammatory mediators by UROtsa cells in response to β-AR activation.

### β-AR Mediated Activation of MAPK Pathway

**Figure 4:**
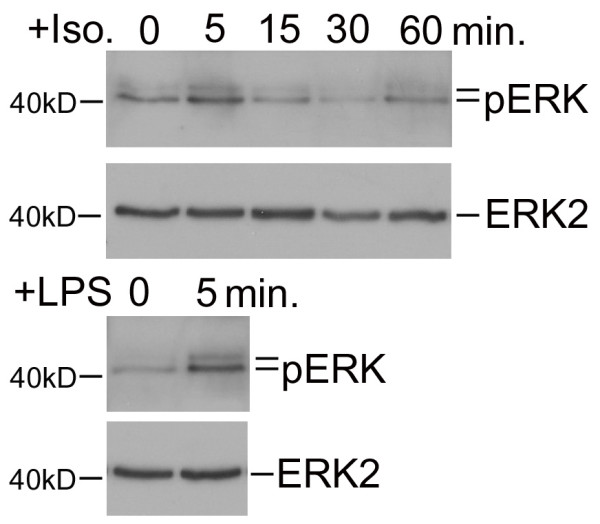
**β-AR mediated activation of the MAPK pathway**. UROtsa cells were incubated with 100 nM isoproterenol or 300 nM LPS for the indicated times, and cell lysates were immunoblotted with anti-pERK to determine the receptor mediated phosphorylation state of ERK or anti-ERK2 to establish the total amount of ERK2 in the samples. As per manufacturer's literature, pERK antibody binds phorphorylated ERK1 and ERK2 in two bands found at 44 and 42 kD, respectively. The ERK2 antibody only binds ERK2 at 42 kD. Peak levels of pERK were observed 5 min after addition of isoproterenol with no significant changes in the total cell lysate levels of ERK2. Densitometric analysis demonstrated a significant 2.4 ± 0.4 fold increase in pERK levels when compared to basal. Similarly, LPS treatment also increased ERK phosphorylation over basal within 5 min without a change in ERK2 production. Values are presented as the mean ± S.E. and the autoradiographs are representative of *n *= 3–10 independent UROtsa cell treatments.

Increased production of inflammatory mediators is associated with induction of the mitogen activated protein kinase (MAPK) signal transduction pathways [14]. Stimulation of β-ARs, although classically linked to cAMP accumulation, has also has been shown to activate pro-inflammatory MAPK pathways [15]. To test whether β-AR stimulation activates intercellular MAPK we assayed isoproterenol treated UROtsa cells for levels of pERK using Western analysis. Cells stimulated with an effective isoproterenol concentration (100 nM) showed significant increases in the amount of pERK 5 min after drug addition (Figure 4). Semi-qualitative analysis revealed a 2.4 ± 0.4 fold increase in pERK levels (*n *= 10) when compared to basal while levels of non-phosphorylated ERK2 remained constant. As a control, UROtsa cells treated with 300 nM LPS showed an increase in ERK phosphorylation after 5 min, which is similar to what was observed using isoproterenol (*n *= 3; Figure 4). These results demonstrate that a MAPK signal transduction pathway is activated by UROtsa cell β-AR stimulation and that ERK phosphorylation precedes transcriptional induction of COX-2 or iNOS.

### ERK Phosphorylation is Independent of cAMP Mediated Activation of PKA

**Figure 5:**
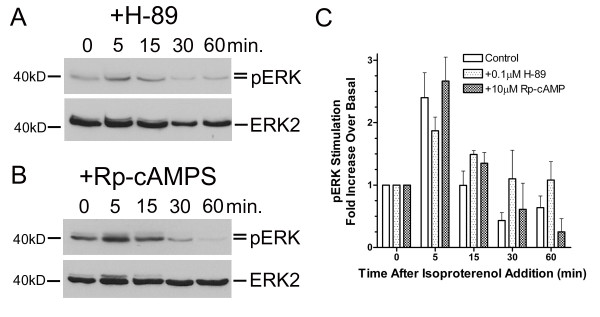
**β-AR stimulation activates the MAPK pathway after treatment with selective PKA inhibitors**. After a 30 min preincubation with 100 nM of the PKA inhibitors H-89 (*panel A*) or 10 μM of Rp-cAMPS (*panel B*), UROtsa cells were stimulated with 100 nM isoproterenol for the indicated times and immunoblotted with anti-pERK or anti-ERK2. Peak levels of pERK were observed within 5 min after the addition of isoproterenol with no changes in the total cell lysate levels of ERK2. Semi-quantitave analysis of the immunoblots revealed a time-dependent increase in ERK phosphorylation (*panel C*). Five minutes after treatment, isoproterenol significantly induced a 1.9 ± 0.4 and 2.6 ± 0.7 fold increase in pERK levels over basal after H-89 or Rp-cAMPS pretreatment, respectively. These peak values are not significantly different than the fold increase over basal for pERK measured in the absence of PKA inhibitor (2.6 ± 0.9). Values are presented as the mean ± S.E. and the autoradiographs are representative immunoblots of *n *= 3–4 independent UROtsa cell treatments.

Increased levels of cAMP are canonically associated with subsequent activation of protein kinase A (PKA). While our results demonstrated a β-AR mediated rise in cAMP production, it remained unclear whether phosphorylation of ERK was dependent upon this cAMP-dependent activation of PKA. Therefore, we studied the effects of UROtsa cell β-AR stimulation after treatment with selective concentrations of the PKA inhibitors H-89 or Rp-cAMPS. Cell pretreatment in serum-free DMEM containing 100 nM of H-89 or 10 μM of Rp-cAMPS did not significantly alter levels of pERK after stimulation with isoproterenol when compared to cells preincubated in the absence of PKA inhibitors (Figure 5). Increases in pERK when compared to basal were still detected following H-89 or Rp-cAMPS pretreatment with peak levels generated at 5 min. Semi-quantitave analysis of the immunoblots revealed a 1.9 ± 0.4 and 2.6 ± 0.7 fold increase in pERK levels over basal after H-89 (*n *= 4) or Rp-cAMPS (*n *= 3) pretreatment, respectively, which are not significantly different than the fold increase for pERK observed without inhibitor pretreatment (2.6 ± 0.9; *n *= 4). Results of these experiments demonstrate that β-AR stimulated MAPK activation in UROtsa cells is not dependent upon generation of cAMP production and subsequent activation of PKA.

### PKA-Independent Production of Inflammatory Mediators

**Figure 6:**
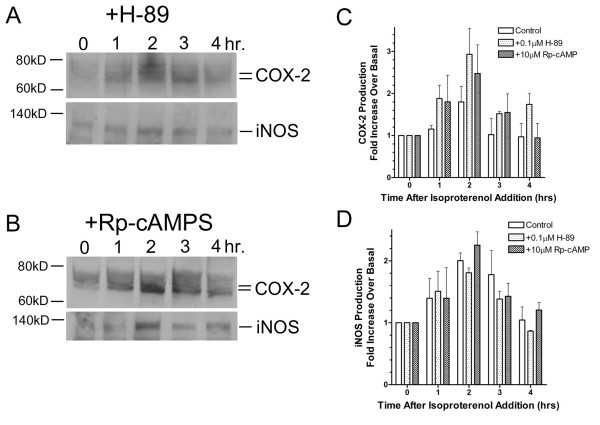
**β-AR stimulated expression of pro-inflammatory mediators occurs through PKA-independent mechanisms**. After a 30 min pre-incubation with 100 nM of H-89 (*panel A*) or 10 μM of Rp-cAMPS (*panel B*), UROtsa cells were stimulated with 100 nM isoproterenol in serum-free DMEM for the indicated times and immunoblotted with anti-COX-2 or anti-iNOS antibody to determine β-AR mediated changes in protein expression. Peak UROtsa cell expression of the pro-inflammatory mediators COX-2 and iNOS was observed 2 hrs after addition of isoproterenol even after pre-incubation with selective PKA inhibitors (*panels C and D*). Levels of COX-2 generated in the presence of H-89 or Rp-cAMPS were significantly increased 3.0 ± 0.4 and 2.5 ± 0.7 fold over basal, respectively. However these isoproterenol induced levels of COX-2 were not significantly different from cells pretreated in the absence of inhibitor (1.9 ± 0.5 fold over basal). Likewise, levels (fold over basal) of iNOS production generated by isoproterenol after H-89 (1.8 ± 0.3) or Rp-cAMPS pretreatment (2.2 ± 0.6) were significantly greater than basal. However these responses were not significantly different from levels observed for isoproterenol induced iNOS production in the absence of PKA inhibitors (2.0 ± 0.7). Values are presented as the mean ± S.E. and the autoradiographs are representative immunoblots of *n *= 3–5 independent UROtsa cell treatments.

Although previous results revealed that MAPK activation is not dependent upon PKA activation, we were interested in whether or not production of inflammatory mediators initiated by β-AR activation also occurred under PKA-independent mechanisms. Pretreatment of UROtsa cells with selective concentrations of H-89 (100 nM) or Rp-cAMPS (10 μM), again did not affect production of COX-2 or iNOS 2 hrs after addition of isoproterenol, when compared to cells pretreated in the absence of inhibitor (Figure 6). In these experiments, levels of COX-2 generated by β-AR activation in the presence of H-89 (*n *= 3) or Rp-cAMPS (*n *= 3) were increased 3.0 ± 0.4 and 2.5 ± 0.7 fold over basal, respectively. This level of COX-2 expression in response to isoproterenol was not significantly different from cells pretreated in the absence of inhibitor (1.9 ± 0.5 fold over basal; *n *= 5). Likewise, levels of β-AR mediated iNOS production after H-89 (1.8 ± 0.3 fold over basal; *n *= 5) or Rp-cAMPS pretreatment (2.2 ± 0.6 fold over basal; *n *= 3) were not significantly different from levels seen in the absence of PKA inhibitors (2.0 ± 0.7 fold over basal; *n *= 3). These results provide evidence for selective production of inflammatory mediators in UROtsa cells through activation of β-ARs that is independent of PKA.

### PKA-Dependent Phosphorylation of the Cyclic AMP-Responsive Element Binding Protein

**Figure 7:**
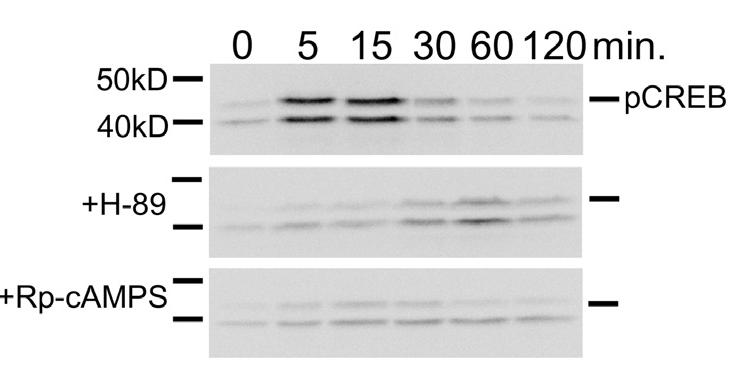
**PKA inhibitors H-89 and Rp-cAMPS block phosphorylation of CREB**. After a 30 min pre-incubation with serum-free DMEM alone or with 100 nM of H-89 or 10 μM of Rp-cAMPS, UROtsa cells were stimulated with 100 nM isoproterenol in serum-free DMEM for the indicated times and immunoblotted with an antibody specific to CREB phosphorylated at S129 and S133 (pCREB). Without inhibitor pretreatment, isoproterenol induced CREB phosphorylation within 5 min. Significant phosphorylation was still observed 15 min. after drug addition with banding intensity patterns similar to basal after 60 min. Preincubation with H-89 or Rp-cAMPS significantly decreased the phosphorylation of CREB at the 5 and 15 min time points in UROtsa cells, demonstrating the effectiveness of PKA inhibition for these compounds. As reported by the antibody suppliers, pCREB is identified as a band running at 43 kD. The second band seen on the autoradiographs represents a previously-reported alternative splice variant of CREB. Autoradiographs are representative immunoblots of *n *= 3 independent cell treatments.

Since our results reveal no changes in the amount of ERK phosphorylation or induction of COX-2 and iNOS after pretreatment with selective concentrations of H-89 or Rp-cAMPS, it was necessary to confirm that these PKA inhibitors were being used effectively in our system. Therefore, we examined the isoproterenol mediated phosphorylation state of the cAMP responsive element binding protein (CREB) in the absence and presence of selective concentrations of H-89 or Rp-cAMPS. CREB is a well characterized transcriptional factor, which is activated by cAMP-dependent PKA phosphorylation of specific serine residues [16]. In our experiments, the isoproterenol induced phosphorylation state of CREB was observed over a 2 hr interval (Figure 7). In the absence of PKA inhibitors, isoproterenol increased the phosphorylation state of CREB within 5 min returning to basal after 60 min (*n *= 3). This time course for CREB phosphorylation is similar to what has been described by others [17]. Pretreatment of UROtsa cells with 100 nM H-89 or 10 μM Rp-cAMPS significantly blocked the isoproterenol mediated CREB phosphorylation (*n *= 3). These results confirm that selective concentrations of H-89 and Rp-cAMPS used for this study can effectively block a cAMP-dependent PKA phosphorylation process. Furthermore, this finding supports our previous observations that the selective production of inflammatory mediators through induction of β-ARs is unrelated to the cAMP-dependent activation of PKA.

## Discussion

This study characterizes a novel role of β-AR signaling in urothelial cells that leads to selective induction of protein products associated with inflammatory responses. Our results demonstrate that a previously described human urothelial cell line expressing functional β-ARs increases production of cAMP, phosphorylated ERK and heightened translation of COX-2 and iNOS in response to agonist activation. β-AR stimulation classically precedes cAMP accumulation, which regulates the activity of PKA leading to phosphorylation of PKA-sensitive substrates. However, phosphorylation of ERK and selective production of inflammatory mediators in UROtsa cells occurs independently of PKA activation, as similar results were observed in the presence of two analogous inhibitors specific for this cAMP-dependent kinase. Effective use of these compounds was confirmed by documenting the inhibition of PKA dependent protein phosphorylation in our same model system. Therefore, functional β-ARs present on these human urothelial cells elicit pro-inflammatory responses by a PKA-independent mechanism.

Previous studies by others have demonstrated the link between activation of MAPK pathways and the induction of inflammatory mediators [14]. In these studies, receptor regulated expression of COX-2 and iNOS was dependent upon the intermediary phosphorylation of ERK. Moreover, β-AR activation, although classically linked to generation of cAMP, has been shown in other studies to influence MAPK activation in a PKA-independent manner [18]. These PKA-independent mechanisms associated with β-AR mediated phosphorylation of ERK have been shown to involve β-arrestin scaffolding complexes [18]. In our studies we show that β-AR mediated ERK phosphorylation in UROtsa cells is independent of active cAMP-dependent PKA. Whether other scaffolding complexes caused by β-AR stimulation in these cells are associated with ERK phosphorylation is currently under investigation by our laboratory.

Despite the fact that a specific etiology has yet to be identified, inflammatory pain is a common mechanism associated with the symptoms of IC [1]. With reference to our human urothelial cell model, we demonstrate an induction of mediators associated with inflammatory pain and bladder hyperexcitability in response to β-AR activation. Clinical correlations have recognized an increased sympathetic innervation as well as elevated catecholamine levels in IC patients when compared to controls [9,10]. Our studies suggest that chronic urothelial β-AR stimulation in these patients may induce COX-2 and iNOS leading to the increased progression of inflammatory pain and bladder hyperexcitability associated with this disease. Induction of COX-2 by bacterial lipopolysaccharide or endogenous cytokines has been shown to elevate prostanoid levels that are linked to the increased vasodilatation, vascular permeability and hyperalgesic responses of inflammation [2]. In other models, induction of iNOS by these same agonists to generate nitric oxide contributes to the nociceptive processing of inflammatory pain [19]. Therefore, we suggest that chronic urothelial β-AR stimulation leading to increased levels of prostaglandins and NO is one potential mechanism of inflammatory pain in IC. Moreover, higher levels of prostanoids and NO may also contribute to the symptomatic increases in urinary frequency and urgency diagnosed in patients with IC [4,8].

Support of this hypothesis has been reported using a mouse model of bladder inflammation in which genes encoding for iNOS and the β_2_-AR subtype were upregulated when compared to control [11]. Interestingly, a significant increase in genomic expression of the β_2_-AR subtype was only observed in a chronic and not an acute bladder inflammation model. Conversely, other investigators have shown using transient application of β-AR agonists that an increase in cAMP is sufficient to generate maintenance levels of NO in primary rat urothelial cells [20]. However, our studies using a human urothelial cell model, demonstrates that cAMP-dependent PKA activation is not necessary to induce inflammatory mechanisms for generating NO. Moreover, generation of homeostatic levels of NO in the rat model was sensitive to Ca^2+ ^indicating that the responsible enzyme was eNOS, although transcriptional message (mRNA) for iNOS was well documented in this same report [20]. This suggests that chronic β-AR stimulation may induce expression of iNOS, which would generate higher levels of NO contributing to the production of inflammatory pain and increased micturition associated with IC.

In our human urothelial cell model we document a β-AR stimulated, PKA-independent signaling pathway that simultaneously increases the expression of two mediators of inflammation, COX-2 and iNOS. In addition, the pathophysiology linked to increased prostaglandin and NO production correlate well with the clinical manifestations associated with chronic inflammatory diseases like IC. Consequently, we believe that UROtsa cells serve as a readily accessible model for studying the β-AR-effector system associated with inflammation in IC. Nonsteroidal anti-inflammatory drugs (NSAIDs), which block the synthesis of prostaglandins by inhibiting COX, are commonly prescribed to relieve discomforts associated with IC. Moreover, NSIADs have been shown to decrease the amount of NO *in vivo *indicating the importance of COX-2 activity in regulating NO production during inflammation [21]. Furthermore, combined pharmacological inhibition of COX-2 and iNOS in a rat model of tonic pain, produces a synergistic antinociceptive effect [22]. This data suggests a common mechanism of action between these two drug classes, however, the associations between COX-2 and iNOS effector systems are currently unknown. Therefore, UROtsa cells represent a unique cell model whereby signal-transduction pathways common to the induction of both COX-2 and iNOS can be investigated. These studies not only may reveal novel targets of inflammatory pain that could be exploited therapeutically, but would increase our understanding of the etiology for general bladder inflammation and hyperexcitability in IC.

## Conclusion

Stimulation of β-ARs expressed on cultured human urothelial cells leads to ERK phosphorylation and production of the pro-inflammatory enzymes. While cAMP levels rise in these cells after β-AR activation, production of COX-2 and iNOS are not dependent upon an increased cAMP regulated PKA activity. Continual initiation of AR function documented for patients diagnosed with IC would likely stimulate urothelial cell inflammatory responses thereby contributing to the etiology of this disease. Our results suggest that by focusing on common urothelial β-AR mediated inflammatory signaling pathways, reasonable pathophysiological mechanisms and potential therapeutic strategies could be developed for chronic inflammatory diseases like IC.

## Methods

### Cell Culture

The immortalized human urothelial (UROtsa) cell line was a gift from Donald Sens (University of North Dakota) and was propagated as previously reported [12]. Briefly, undifferentiated UROtsa cells were grown to confluence in serum-containing Dulbecco's Modified Eagle's Medium (DMEM) under standard cell culture conditions. Confluent UROtsa cells were washed in serum-free DMEM and pre-incubated with or without inhibitors protein kinase A (PKA) inhibitors 1 hr before addition of the selective β-AR agonist, isoproterenol. The PKA inhibitors H-89 (Sigma, St. Louis, MO) was used at a final concentration of 100 nM, while Rp-cAMPS (BioLog, Bremen, Germany) was used at 10 μM. Unless noted otherwise, isoproterenol (Sigma, St. Louis, MO) was added to cells at a final concentration of 100 nM. As a nonspecific initiator of inflammation control, cells were incubated with 300 nM lipopolysaccharide (CalBioChem, La Jolla, CA).

### Membrane Preparation

A crude cell membrane preparation was prepared as previously described [23]. Briefly, UROtsa membranes were prepared by transferring suspended cells to a 50 mL conical tube and twice washing by centrifugation at 1000 × *g *using cold Hank's balance salt solution (HBSS). The intact cell pellet was resuspended in 10 mL of 0.25 M sucrose containing 10 μg/mL bacitracin, 10 μg/mL benzamidine, 10 μg/mL leupeptin, and 20 μg/mL phenylmethysulfonylfluoride. The cells were disrupted by freezing followed by Dounce homogenization of the thawed suspension using 20 strokes from a loose fitting (B) pestle. This mixture was then centrifuged at 1260 × *g *for 5 min at 4°C. Buffer A (20 mM HEPES, pH 7.5, 1.4 mM EGTA, 12.5 mM MgCl_2_) was added to the supernatant and centrifuged again at 30,000 × *g *for 15 min at 4°C. The resultant pellet was kept, resuspended in buffer A then centrifuged once more at 30,000 × *g *for 15 min at 4°C. The final crude membrane pellet was resuspended in buffer A containing 10% glycerol and stored in aliquots at -70°C until used for radioligand binding. Protein concentrations were measured using the method of Bradford [24].

### Radioligand Binding

The radioligand binding protocol used for this study was performed as previously described [13]. Briefly, the density of expressed β-ARs on UROtsa cells was determined by saturation binding experiments using the nonselective β-AR antagonist ^125^I-CYP as the radiolabel (NEN Life Sciences, Boston, MA). Crude UROtsa cell membranes were allowed to equilibrate at 37°C with increasing concentrations of ^125^I-CYP (5–600 pM) in a 0.25 mL total volume of buffer A using 10^-5 ^M propranolol to determine non-specific binding. Binding was stopped by filtering the membranes though Whatman GF/C glass fiber filters, followed by 5 – 5 mL washes with cold buffer A to remove any unbound drug. Amounts of total and non-specific radiolabel bound to cell membranes were calculated from radioactive counts remaining on the glass fiber filters. From the plotted saturation hyperbola, β-AR density (B_max_) and the equilibrium dissociation constant (K_d_) of ^125^I-CYP for specific UROtsa cell binding sites were calculated using iterative non-linear regression analysis [25].

### cAMP Assay

Confluent UROtsa cells used for the quantification of cAMP were treated in serum-free DMEM containing 1 mM 1-methyl-3-isobutylxanthine (IBMX) to inhibit phosphodiesterase. After 30 min of isoproterenol treatment, cells were lysed using 0.1 M HCl and collected for determination of cAMP production according to the Biotrak Assay System protocol (Amersham, Buckinghamshire, UK). Briefly, ^3^H-cAMP added to cell lysates was used to compete with endogenous cAMP for binding to a specific cAMP-binding protein. ^3^H-cAMP levels were then counted by liquid scintillation and related to endogenously generated cAMP by comparison with known standards. The concentration of isoproterenol that caused a half-maximal generation of cAMP (EC_50_) was calculated from non-linear regression analysis using Prism 4 (Graphpad Software, San Diego, CA).

### Western Hybridization

After an appropriate period of time, treated cells were lysed using a modified RIPA buffer (150 mM NaCl, 10 mM Tris, pH 7.2, 0.1% sodium dodecylsulphate, 1.0% Triton X-100, 1.0% sodium deoxycholate, 5 mM EDTA, 1.0% protease inhibitor cocktail; Sigma, St. Louis, MO). Total cell lysate protein concentrations were estimated using Bradford protein assay reagent (Bio-Rad, Hercules, CA) before lysates were resolved by SDS-PAGE and transferred to nitrocellulose membranes. Protein expression was measured by 4°C overnight immunoblotting with diluted antibodies: extracellular signal-regulated kinase 2 (mouse monoclonal ERK2, 1:1000; Santa Cruz Biotechnology, Santa Cruz, CA), phosphorylated ERK1/2 (mouse monoclonal pERK, 1:500; Santa Cruz Biotechnology), COX-2 (goat polyclonal, 1:500; Santa Cruz Biotechnology) iNOS (rabbit polyclonal, 1:500; Santa Cruz Biotechnology) and phosphorylated CREB (rabbit polyclonal, 1:1000; AbCam, Cambridge, MA). After washing, membranes were incubated at 25°C for 90 min with diluted horseradish peroxidase-linked secondary antibody (1:1000–5000). Bound antibody was visualized by the Supersignal West Pico chemiluminescent system (Pierce, Rockford, IL) and exposed to radiographic film. Developed films were subsequently photographed and protein band intensity was estimated by semi-quantitative densitometric methods using LabWorks v.4.5 (UVP, Upland, CA). Protein levels are presented as the mean fold increase in pixel intensity over control, plus or minus the standard error for *n *experiments. Differences between control and drug treated groups was determined using a paired one-tailed Student's *t *test with a *p *< .05 level of probability accepted as significant. Equal protein loading was confirmed by Ponceau-S staining of nitrocellulose membranes.

## Competing interests

The author(s) declare that they have no competing interests.

## Authors' contributions

EBH performed all of the experiments and was responsible for acquisition, analysis and interpretation of the data. JMP provided assistance for the western blot analysis as part of a NSF funded summer fellowship. JEP conceived, monitored, and coordinated the experimental design. Both EBH and JEP contributed equally to the writing of this manuscript.
